# Clinical impacts of the concomitant use of L-asparaginase and total parenteral nutrition containing L-aspartic acid in patients with acute lymphoblastic leukemia

**DOI:** 10.3389/fnut.2023.1122010

**Published:** 2023-04-03

**Authors:** Minoh Ko, Myeong Gyu Kim, Sung-Soo Yoon, In-Wha Kim, Sung Yun Suh, Yoon-Sook Cho, Jung Mi Oh

**Affiliations:** ^1^College of Pharmacy and Research Institute of Pharmaceutical Sciences, Seoul National University, Seoul, Republic of Korea; ^2^Department of Pharmacy, Seoul National University Hospital, Seoul, Republic of Korea; ^3^College of Pharmacy and Graduate School of Pharmaceutical Sciences, Ewha Womans University, Seoul, Republic of Korea; ^4^Department of Internal Medicine, Seoul National University Hospital, Seoul, Republic of Korea; ^5^Cancer Research Institute, Seoul National University, Seoul, Republic of Korea

**Keywords:** L-asparaginase, L-aspartic acid, total parenteral nutrition, interaction, acute lymhoblastic leukaemia, effectiveness, safety, propensity score matching

## Abstract

**Introduction:**

L-asparaginase (ASNase) depletes L-asparagine and causes the death of leukemic cells, making it a mainstay for the treatment of acute lymphoblastic leukemia (ALL). However, ASNase's activity can be inhibited by L-aspartic acid (Asp), which competes for the same substrate and reduces the drug's efficacy. While many commercially used total parenteral nutrition (TPN) products contain Asp, it is unclear how the concomitant use of TPNs containing Asp (Asp-TPN) affects ALL patients treated with ASNase. This propensity-matched retrospective cohort study evaluated the clinical effects of the interaction between ASNase and Asp-TPN.

**Methods:**

The study population included newly diagnosed adult Korean ALL patients who received VPDL induction therapy consisting of vincristine, prednisolone, daunorubicin, and *Escherichia coli* L-asparaginase between 2004 and 2021. Patients were divided into two groups based on their exposure to Asp-TPN: (1) Asp-TPN group and (2) control group. Data, including baseline characteristics, disease information, medication information, and laboratory data, were collected retrospectively. The primary outcomes for the effectiveness were overall and complete response rates. Relapse-free survival at six months and one year of treatment were also evaluated. The safety of both TPN and ASNase was evaluated by comparing liver function test levels between groups. A 1:1 propensity score matching analysis was conducted to minimize potential selection bias.

**Results:**

The analysis included a total of 112 ALL patients, and 34 of whom received Asp-TPN and ASNase concomitantly. After propensity score matching, 30 patients remained in each group. The concomitant use of Asp-TPN and ASNase did not affect the overall response rate (odds ratio [OR] 0.53; 95% confidence interval [CI] = 0.17–1.62) or the complete response rate (OR 0.86; 95% CI = 0.29–2.59) of the ASNase-including induction therapy. The concomitant use of Asp-TPN and ASNase also did not impact relapse-free survival (RFS) at six months and one year of treatment (OR 1.00; 95% CI = 0.36–2.78 and OR 1.24; 95% CI, 0.50–3.12, respectively). The peak levels of each liver function test (LFT) and the frequency of LFT elevations were evaluated during induction therapy and showed no difference between the two groups.

**Conclusion:**

There is no clear rationale for avoiding Asp-TPN in ASNase-treated patients.

## 1. Introduction

Although survival outcomes have improved dramatically in pediatrics with acute lymphoblastic leukemia (ALL), cure rates for adult ALL patients have been estimated to be only 40% (1, 2). According to several studies, the initial response affects the prognosis of ALL. High levels of minimal residual disease (MRD) have been found to be associated with decreased relapse-free survival (RFS) after induction therapy. Thus, any potential factor that could affect the initial response of induction treatment needs to be reduced to maximize long-term responses. For newly diagnosed adult ALL patients, the regimen consisting of vincristine, prednisolone, daunorubicin, and L-asparaginase (VPDL) is used as induction therapy (3).

**Figure 1:**
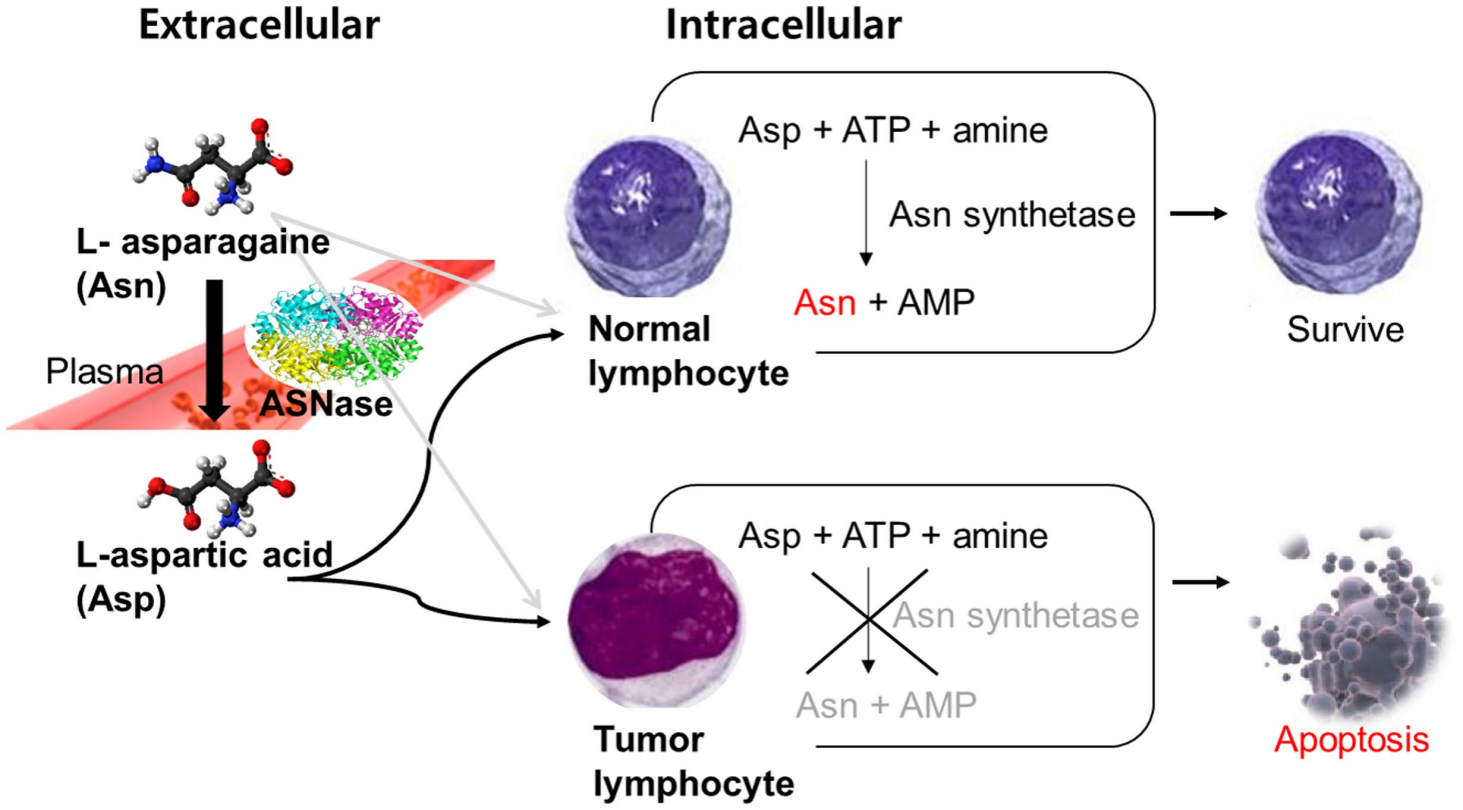
Mechanism of L-asparaginase in tumor cells. Asn, L-asparagine; ASNase, Asparaginase; Asp, L-aspartic acid; ATP, Adenosine triphosphate; AMP, Adenosine monophosphate.

L-asparaginase (ASNase) is an enzyme that catalyzes the hydrolysis of L-asparagine into L-aspartic acid (Asp). In blood and cerebrospinal fluid, ASNase leads to a rapid lowering of the levels of asparagine, an amino acid that is crucial for cell growth (4–7). While normal cells can synthesize asparagine, leukemic lymphoblasts lack asparagine synthetase and are incapable of *de novo* asparagine synthesis, which makes them dependent on the exogenous supply of asparagine. ASNase results in apoptosis of tumor cells by draining all circulating asparagine as shown in Figure 1 (8). Previous binding and inhibition studies (9–11) demonstrated the interaction between this enzyme drug and its product, Asp. One study found that Asp competitively inhibits ASNase with the inhibition constant of 80 μM (9). Based on these studies, a way to chemically remove Asp from ASNase was suggested to maintain the activity of ASNase and to prevent this interaction. Now, commercially supplied ASNase does not contain Asp.

Meanwhile, ALL patients undergoing chemotherapy suffer from malnutrition due to mucositis and anorexia caused by chemotherapy, which contributes to the mortality rate of cancer patients (12–16, 26). Total parenteral nutrition (TPN) provides a way to deliver nutrients directly into the bloodstream to help maintain adequate nutrition in these patients, who may not be able to consume enough food orally. TPN products contain several kinds of amino acids, and Asp is included among them. Asparagine, on the other hand, is excluded from the TPN products because normal cells can synthesize asparagine *via* the actions of asparagine synthetase. Because Asp-containing TPN (Asp-TPN) formulations are being concomitantly used with ASNase, we are concerned that Asp in TPN may affect the clinical response of ASNase. However, whether the interaction between ASNase and Asp-TPN would impact the clinical outcomes in newly diagnosed ALL patients has never been studied. As previously mentioned, achieving complete remission (CR) with induction therapy is important for ALL prognosis. If an amino acid known to affect the activity of the cornerstone of induction therapy is being administered concurrently with TPN, clinical analysis of the interaction is significant. Accordingly, we compared the response, relapse, and safety between the control and Asp-TPN groups to ensure ASNase and TPN can be used concomitantly in ALL patients.

## 2. Materials and methods

### 2.1. Study population

Newly diagnosed adult Korean ALL patients were included who administered *Escherichia coli* ASNase-including VPDL induction therapy (vincristine 1.5 mg/m^2^ iv, maximum 2 mg on days 1, 8, 15, and 22, prednisolone 40 mg/m^2^ or 60 mg/m^2^ po on days 1–22 with tapering off, daunorubicin 45 mg/m^2^, 60 mg/m^2^, or 90 mg/m^2^ iv on day 1–3, and ASNase 6,000 IU/m^2^ iv, maximum 10,000 IU on day 12–21) at Seoul National University Hospital (SNUH) between the years 2004 and 2021. Patients who had been diagnosed with other hemato-oncological diseases and exposed to other chemotherapy regimens were excluded from this study. Additionally, we excluded those patients receiving ASNase doses less than 30,000 IU/m^2^ (50% of the normal dose). The patients were followed up for relapse until a year had passed from the first remission. Patients who met inclusion criteria were then divided into two groups based on their exposure to Asp-TPN: (1) Asp-TPN group and (2) control group. The study was approved by the Institutional Review Board of Seoul National University Hospital Clinical Research Institute (IRB No. 2103-076-1204).

### 2.2. Data collection

All data, including baseline characteristics, disease, medication information, and laboratory data, were collected retrospectively from electronic medical records. Data including age, sex, body mass index (BMI), and body surface area (BSA) were collected as baseline characteristics. Types of diets along with methods of nutrition during ASNase administration and performance status (PS) based on the Eastern Cooperative Oncology Group (ECOG) score at the beginning of induction therapy were also obtained (17). To classify the risk category of ALL following National Comprehensive Cancer Network (NCCN) guidelines (3), white blood cell (WBC) count (×10^9^/L), bone marrow blast (%), immunophenotype, cytogenetic and molecular subtypes, and extramedullary or other organ involvement were gathered. As previous chemotherapy could affect patients’ sensitivity to chemotherapy, comorbidities were collected and divided into oncological and non-oncological diseases. The medication information obtained for each subject included the dose and dosing schedule of VPDL induction therapy, as well as the types and doses of TPN formulations and any concomitant drug therapies. To analyze the clinical effects of VPDL induction therapy, the laboratory data were collected from the first day of admission to the day of discharge after completion of VPDL induction therapy. The following data were included: absolute neutrophil count (ANC) (×10^9^/L), platelet count (×10^9^/L), and blast (%) of peripheral blood on the day of bone marrow exam and during follow-up visit after discharge, liver function tests (LFTs) including total bilirubin, aspartate aminotransferase (AST), alanine aminotransferase (ALT), alkaline phosphatase (ALP), and gamma-glutamyl transferase (GGT).

### 2.3. Outcomes

The effectiveness of concomitant use of Asp-TPN and ASNase was primarily compared by determining the overall and complete response rates (ORR and CRR, respectively) between the Asp-TPN and the control groups on the day that the bone marrow examination was conducted. The ORR of VPDL induction therapy was defined as a combination of complete response (CR) and CR with incomplete count recovery (CRi) in accordance with NCCN guidelines. Other measures of effectiveness included RFS, which followed the definition made by the Berlin-Frankfurt-Münster (BFM) and Children’s Oncology Group (COG) groups (18). Very early and early relapse rate were evaluated by the RFS rate (%) at 6 months and 1 year of treatment, respectively.

For safety evaluation of both TPN and ASNase, we specifically compared the levels of LFTs between two groups at baseline and after VPDL induction. The peak levels of LFTs were categorized according to the Common Terminology Criteria for Adverse Events (CTCAE version 4.03, Bethesda, MD). Along with absolute peak levels of LFT values, the percentage of patients with grade 2 or more adverse events associated with the LFTs increase was compared between the groups.

### 2.4. Statistical analysis

Based on the results of the Shapiro–Wilk test, continuous variables were compared using a t-test or Mann–Whitney U test and presented as either mean (standard deviation [SD]) or median (range). Categorical data were compared by Pearson’s chi-squared or Fisher’s exact test and described as frequency. Survival analysis was performed using the Kaplan–Meier method and compared using log-rank analysis. To minimize the potential selection bias, a 1:1 propensity score matching analysis was conducted using the nearest-neighbor method with a caliper of 0.20. The propensity score was estimated by using the following predictors: (1) age, (2) sex, (3) BSA, (4) BMI, (5) risk category for ALL, (6) the total dose of ASNase, (7) total administered days of ASNase, (8) performance status on admission, (9) central nervous system (CNS) involvement at diagnosis, (10) nutrition risk, (11) albumin on the first day of chemotherapy, (12) dose modification of VPDL induction, and (13) previous history of the disease. All analyses were performed using the R-software (R for Windows 4.2.1; The R Foundation for Statistical Computing, Vienna, Austria) with a significance level of 0.05. MatchIt package for the R-software was used for propensity score-based matching.

## 3. Results

### 3.1. Patient characteristics

**Figure 2:**
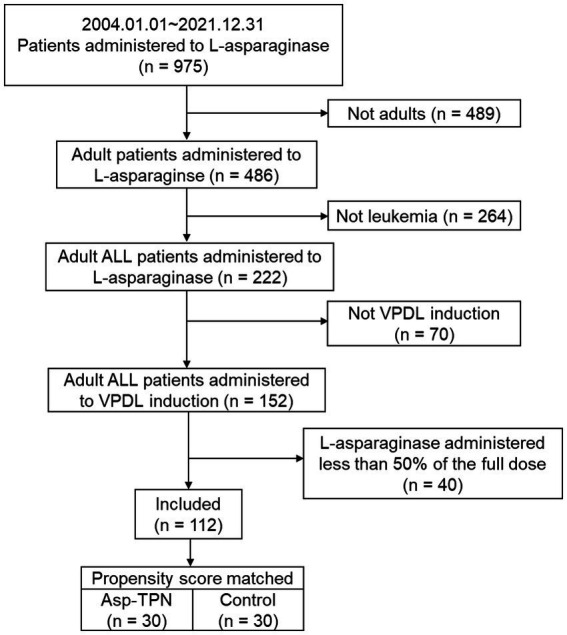
Flowchart of the study population. ALL, acute lymphoblastic leukemia; VPDL induction, Vincristine/Prednisolone/Daunorubicin/L-asparaginase induction regimen; Asp-TPN, aspartic acid-containing total parenteral nutrition.

**Figure 3:**
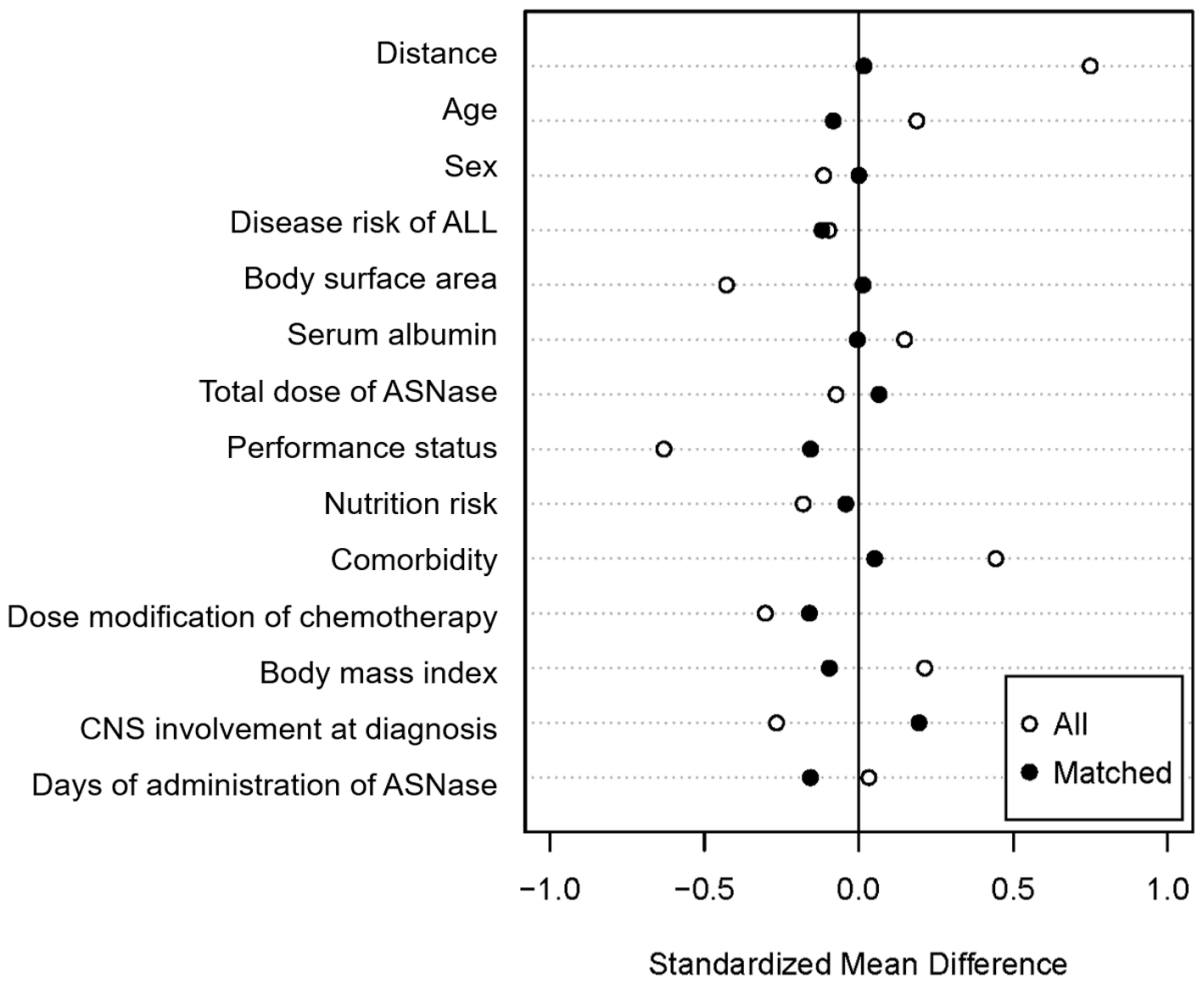
Covariate balance after propensity score matching. ALL, acute lymphoblastic leukemia; ASNase, L-asparaginase; CNS, Central nervous system.

A total of 112 newly diagnosed ALL patients who received VPDL induction therapy between 2004 and 2021 were included in the study (Figure 2). Among 112 included patients, 33 patients (29.5%) were concomitantly treated with Asp-TPN at least once during ASNase therapy. The median duration of co-administration of Asp-TPN and ASNase was 4 days (range 1–10 days), and the median dose of Asp administered by TPN was 1.50 (0.5–3.20) g/day. Before group matching, the weight of the Asp-TPN user group was lower than the control group (65.3 kg versus 68.0 kg; *p* = 0.08). To minimize the effect of baseline nutrition status on the outcomes, propensity score matching was conducted, and 30 patients from the Asp-TPN group were matched to 30 patients in the control group. The similar weights of both groups (Asp-TPN group versus Control group: 61.0 kg versus 62.0 kg; *p* = 0.86) showed the balance between the groups (Figure 3).

Overall, baseline characteristics, including nutrition risk, BMI, LFTs and albumin of matched cohorts were similar between groups (Table 1). Factors that could affect ALL treatment outcomes, including disease risk, ASNase dose, CNS involvement, and prophylactic use of intrathecal methotrexate were comparable between groups. A similar proportion of high-risk ALL patients were included in each group (63.3% in the Asp-TPN group and 53.3% in the control group; *p* = 0.62). The number of gene mutation BCR–ABL positive patients taking tyrosine kinase inhibitors (TKIs), which are known to be critical factors for treatment outcomes of ALL, was similar between groups (nine people in the Asp-TPN group versus seven people in the control group; *p* = 0.78). At the time of admission, there were slightly more patients with ECOG scores of 3 or more in the Asp-TPN group, but the difference was not statistically significant (11 versus 9; *p* = 0.80). When it came to comorbidities, two patients in the Asp-TPN group and one patient in the control group had received treatment for solid cancers (*p* = 0.49). Additionally, no differences were noted in the total doses of ASNase, vincristine, daunorubicin, and prednisolone between the Asp-TPN and control groups.

**Table 1:** Baseline characteristics of the propensity score-matched patients.

	Asp-TPN group (*n* = 30)	Control group (*n* = 30)	*p*-value
Age (years)^a^	41 (26–50)	42 (26–55)	0.98
Sex (male)	13 (43.3%)	13 (43.3%)	1.00
BMI (kg/m^2^)^a^	22.2 (16.6–32.6)	22.6 (16.9–30.5)	0.53
BSA (m^2^)^a^	1.68 (1.44–2.22)	1.69 (1.42–1.96)	0.96
Albumin (g/dL)^a^	3.9 (2.9–4.4)	3.8 (2.9–4.9)	0.79
Disease history			0.46
Oncologic disease	3 (10.0%)	1 (3.3%)	
Other than malignancy	7 (23.3%)	10 (33.3%)	
Performance status (ECOG score)			0.41
2	10 (33.3%)	15 (50.0%)	
≥3	11 (36.7%)	9 (30.0%)	
CNS involvement	1 (3.3%)	0 (0%)	1.00
Administered day of ASNase^a^	10 (6–10)	10 (7–10)	0.47
Disease risk of ALL			0.62
Standard	14 (46.7%)	11 (36.7%)	
High / Very high	16 (53.3%)	19 (63.3%)	
Total ASNase dose (×1000IU/m^2^)^a^	6.0 (3.6–6.6)	6.0 (4.2–6.0)	0.47
Nutritional risk (severe)	10 (33.3%)	11 (36.7%)	0.76
Dose modification	14 (46.7%)	17 (56.7%)	0.61
Liver function tests^a^			
Total bilirubin (mg/dL)	0.75 (0.3–16.7)	0.7 (0.5–2.1)	0.46
AST (IU/L)	24.5 (9–340)	27.5 (10–103)	0.92
ALT (IU/L)	21 (6–138)	27 (10–257)	0.09
ALP (IU/L)	83.5 (48–888)	71 (16–246)	0.15

a Median (min-max).

ALL, acute lymphoblastic leukemia. ASNase, L-asparaginase; Asp-TPN, aspartic acid-containing total parenteral nutrition; ALP, alkaline phosphatase. ALT, alanine aminotransferase; AST, aspartate aminotransferase. BMI, body mass index. BSA, body surface area. CNS, central nervous system. ECOG, the Eastern Cooperative Oncology Group.

### 3.2. Outcomes

#### 3.2.1. Effectiveness

Two patients in the Asp-TPN group and one subject in the control group died due to sepsis before the response to induction therapy could be analyzed and were therefore, excluded when evaluating the response. Both the ORR and the CRR showed no difference between the two groups (ORR, 67.9% versus 79.3%; Odds ratio [OR] 0.53; 95% confidence interval [CI] 0.17–1.62; *p* = 0.65 and CRR, 34.5% versus 35.7%; OR 0.86; 95% CI 0.29–2.59; *p* = 1.00), corresponding to Asp-TPN group versus Control group (Table 2).

**Table 2:** Response rates according to the concomitant use of total parenteral nutrition containing L-aspartic acid.

Asp-TPN group (*n* = 30)	Control group (*n* = 30)	OR [95% CI]	*p*-value
Overall response, *n* (%)			
19 (67.9%)	23 (79.3%)	0.53 [0.17–1.62]	0.65
Complete response, *n* (%)			
9 (34.5%)	10 (35.7%)	0.86 [0.29–2.59]	1.00

Asp-TPN, aspartic acid-containing total parenteral nutrition; CI, confidence interval; OR, odds ratio.

**Figure 4:**
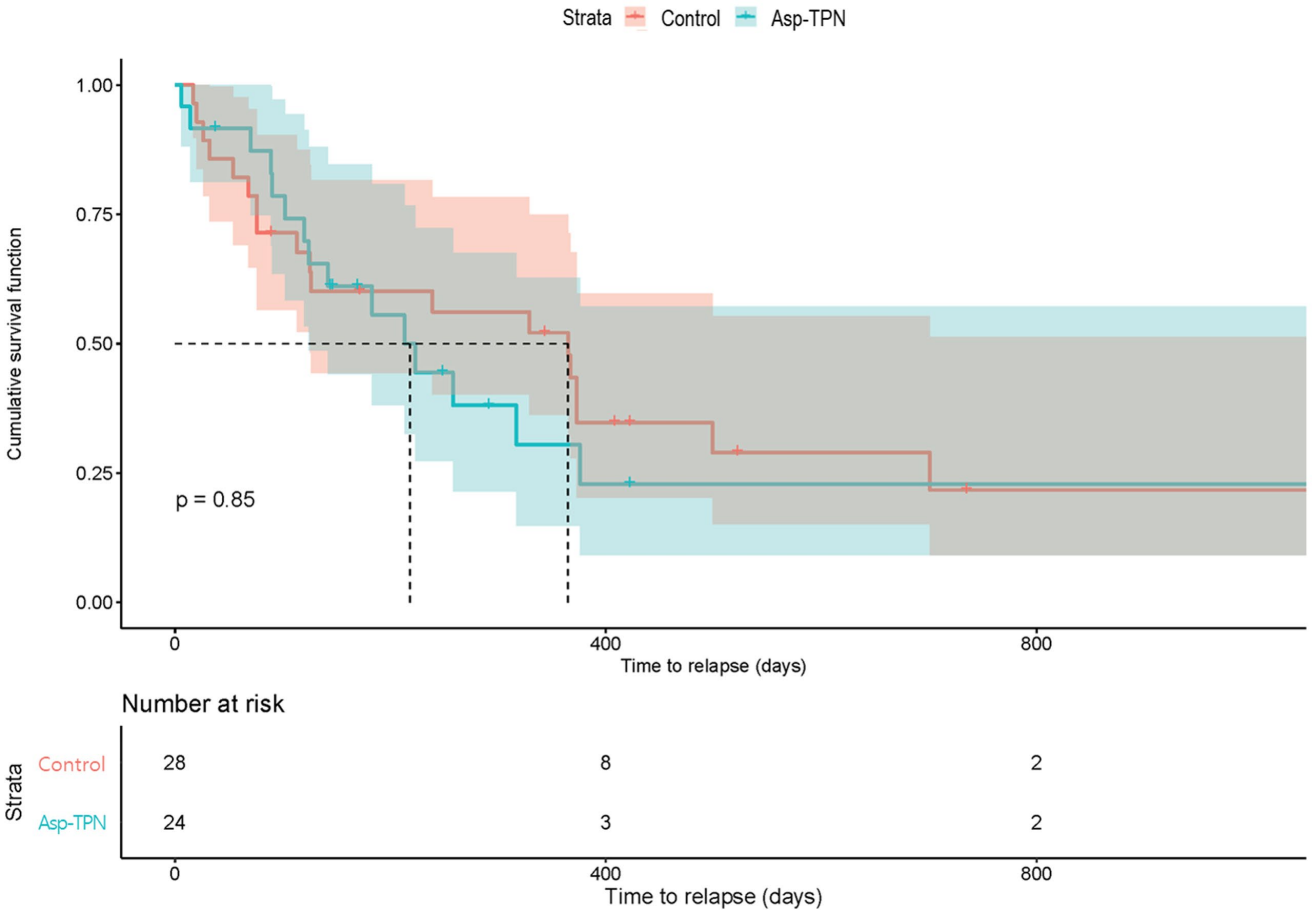
Relapse-free survival according to the concomitant use of total parenteral nutrition containing L-aspartic acid. Asp-TPN, aspartic acid-containing total parenteral nutrition.

We evaluated the impact of Asp-TPN on the relapse of ALL based on 6-month and 1-year RFS. No difference was found in the 6 month RFS between the two groups (OR, 1.00; 95% CI 0.36–2.78) as shown in Figure 4. 1 year after the start of the induction therapy, the concomitant use of ASNase and Asp-TPN led to an increase in the risk of early relapse (OR 1.24; 95% CI 0.50–3.12), and yet the OR did not reach statistical significance (*p* = 0.85, Figure 4).

#### 3.2.2. Safety

As shown in Table 3, no statistically significant differences in absolute peak levels of total bilirubin, aminotransferases, ALP, and GGT between groups while receiving the treatments were found. The median peak levels of total bilirubin, ALT, and GGT were above the upper limits of the normal range. The frequency of grade ≥ 3 LFT elevation was comparable in general. As Table 4 shows, the most common grade ≥ 3 adverse event was blood bilirubin increased (30% versus 26.7% in the Asp-TPN versus the control group). The levels of LFTs increased within 10 days (9 [3–18] versus 7 [1–21], Asp-TPN versus control) after VPDL induction therapy had started. The peak levels of GGT were only available from 19 and 23 patients from the Asp-TPN and control groups, respectively, because GGT levels were only measured when patients showed increased levels in aminotransferases. It explains why the minimum peak levels were above the lower limits of the normal ranges in both groups.

**Table 3:** Peak level of liver function test (LFT) results.

	Asp-TPN group (*n* = 30)	Control group (*n* = 30)	*p*-value
T.bil (mg/dL)^a^	2.5 (1.9–3.7)	2.2 (1.7–4.0)	0.46
AST (IU/L)^a^	41.5 (23–160)	47.0 (28–107)	0.85
ALT (IU/L)^a^	91.5 (48–158)	87.5 (53–204)	0.88
ALP (IU/L)^a^	123.5 (73–198)	113 (90–298)	0.46
GGT (IU/L)^a^^,^^b^	196 (65–291)	133 (70–299)	1.00

a Median (min-max).

b Only 19 of Asp-TPN group and 23 of control group had GGT results.

ALP, alkaline phosphatase; ALT, alanine transaminase; AST, aspartate transaminase; GGT, gamma-glutamyl transferase; T.bil, total bilirubin; Asp-TPN, aspartic acid-containing total parenteral nutrition.

**Table 4:** The frequency of Grade 2 or more adverse events associated with LFT increases.

	Asp-TPN group (*n* = 30)	Control group (*n* = 30)	*p*-value
T.bil increased			
Grade ≥ 2	21 (70.0%)	25 (83.3%)	0.36
Grade ≥ 3	9 (30.0%)	8 (26.7%)	1.00
AST increased			
Grade ≥ 2	6 (20.0%)	8 (26.7%)	0.76
Grade ≥ 3	4 (13.3%)	6 (20.0%)	0.73
ALT increased			
Grade ≥ 2	12 (40.0%)	10 (33.3%)	0.79
Grade ≥ 3	8 (26.7%)	7 (23.3%)	1.00
ALP increased			
Grade ≥ 2	8 (26.7%)	4 (13.3%)	0.33
Grade ≥ 3	2 (6.7%)	0 (0%)	0.49
	**Asp-TPN group (*n*=19** ^**a**^ **)**	**Control group (*n*=23** ^**a**^ **)**	*p*-value
GGT increased			
Grade ≥ 2	9 (47.4%)	13 (56.5%)	0.78
Grade ≥ 3	4 (21.1%)	5 (21.7%)	1.00

a Only 19 of Asp-TPN group and 23 of control group had GGT results.

ALP, alkaline phosphatase; ALT, alanine transaminase; AST, aspartate transaminase; GGT, gamma-glutamyl transferase; T.bil, total bilirubin; Asp-TPN, aspartic acid-containing total parenteral nutrition.

## 4. Discussion

In this retrospective cohort study, the clinical effects of concomitant administration of Asp-TPN formulation and ASNase were evaluated for the first time. Overall, concurrent infusion of Asp-TPN with ASNase did not affect the response of the induction therapy or the very early relapse rate in patients with ALL.

It was imperative to investigate this topic for three primary reasons. To begin with, despite previous *in vitro* studies suggesting the interaction between ASNase and Asp, no study has evaluated whether Asp-TPN would clinically impact the effectiveness of ASNase or not in ALL patients. Furthermore, the new evidence was necessary to decide on the optimized TPN formulation that had minimal interactions with the chemotherapy in ALL patients. Since Asp plays a role in hormone production and release, and helps the nervous system function normally, it is contained in many commercial TPN formulations used for cancer patients. However, no evidence is available on the effectiveness of concomitant administration of Asp-TPN formulations for patients treated with ASNase. In this study, 82.5% of TPN users received Asp-containing formulations. All TPN formulations for patients were ordered by physicians without any preference, which minimize potential bias in the selection of TPN. By conducting this research, we aimed to provide evidence-based recommendations in selecting the appropriate TPN for patients undergoing induction therapy. To determine the optimal TPN formulation for patients receiving ASNase in clinical practice, further studies with larger sample sizes, longer follow-up periods, and well-defined prospective protocols are needed to validate the findings of this study. Moreover, evaluating any factors that would alter the effectiveness of the chemotherapy would help improve the survival rates of patients with ALL.

Our study showed that the concomitant use of ASNase and potential enzymatic inhibitor Asp-TPN formulation did not have an impact on the response and relapse rates of ASNase-including induction therapy, which is inconsistent with previous *in vitro* studies. Asp has been reported to competitively inhibit ASNase in the 6–50 μM range with an inhibition constant (K_i_) of 80 μM (9, 19). The median plasma concentration of Asp in healthy adults was reported as 6 μM (20). Although limited studies on the changes in amino acid concentration after parenteral nutrition are available, a previous study (21) showed that Asp concentration rapidly increased to about 20 μM after TPN administration, a finding that shows the possibility of the interaction between TPN and ASNase. However, the level of enzyme inhibition may vary depending on several factors, such as metal ions, pH, and concentration of the substrates in the mixture, and these factors may be the reason the results of this study were different from those of *in vitro* studies. Additionally, for predicting inhibitory drug–drug interactions, it is necessary to consider the ratio of the concentration of the inhibitor to its inhibitory constant and the ratio of area under the curve (AUC) comprehensively (22). Although not presented in the current study, we also analyzed a dose-dependent relationship between the amount of Asp in the TPN and the response outcomes with no association between these two parameters. According to the results, the concomitant use of ASNase and Asp-TPN was associated with an increased risk of early relapse. However, the observed increase in risk was not statistically significant, with a *p*-value of 0.85. As very early relapse is critical for the prognosis of ALL, further studies considering comprehensive factors with the large number of populations are expected to clarify the relationship between the concomitant use of ASNase and Asp-TPN and the risk of early relapse of ALL.

In this study, we specifically evaluated LFT elevation, which is the most common toxicity of both TPN and ASNase. (23, 24). Although TPN causes a transient increase in aminotransferase concentrations during the first 1 to 3 weeks of TPN, patients receiving long-term TPN may develop TPN-induced liver disease as suggested in previous reports (22, 25). The median duration of TPN administration in our study was less than a week. Thus, this such short-term TPN use during 28-day VPDL induction therapy is unlikely to aggravate ASNase-associated hepatotoxicity. By comparing the frequency and severity of LFT elevation in both groups, we aimed to determine if combination treatment had a higher risk of liver toxicity. Monitoring other ASNase-induced complications such as pancreatitis and disseminated intravascular coagulation (DIC) is important in evaluating the safety of the treatment. However, due to the retrospective nature of the study, it was not possible to include these in the analysis, since triglycerides, or markers for other rare complications were not routinely checked unless patients showed signs or symptoms. A prospective study design with routine monitoring and systemic recording of these parameters would provide more comprehensive understanding of the safety profile. Nevertheless, this study is valuable as it represents an initial attempt for safety evaluation of Asp-TPN in ASNase-treated ALL patients.

Several study limitations should be discussed. First, a limited number of patients in each matched cohort was found as the study was conducted in a single center. According to the latest national cancer statistics in Korea, ALL is a rare disease as it comprises only 1.5% of the total number of new cancer cases. Another reason for the limited number of patients is due to matching process, which enabled the research to minimize the selection bias. Furthermore, due to its retrospective nature, the study had a lack of information on concentrations of amino acid and activity of ASNase in the body that could support our findings. Moreover, gathering information on the amount of dietary intake was limited, but all patients received a low-bacteria diet provided by the hospital. Not all patients in the control group received TPN. However, the authors tried to control the potential bias by propensity score matching including factors that could affect the nutrition status. Also, most of the reason to initiate TPN therapy was to prevent mild, rather than severe, malnutrition caused by mucositis. Additionally, as pediatric patients have distinctive pharmacokinetics and pharmacodynamics, extrapolating the findings from adult patients to pediatric population should be careful. ALL is the most common malignancy in pediatric patients. Therefore, further studies specifically designed for pediatric population is needed in the future.

This study is the first that evaluates the clinical outcomes of the concomitant use of ASNase and Asp-TPN formulations. There were no differences in response rates and relapse-free survival between Asp-TPN and control groups. Peak levels of LFTs and the frequency of LFTs increases were also similar. Based on the results of the current study, Asp-TPN can be administered without concern in ALL patients receiving ASNase as induction therapy.

## Data availability statement

The datasets generated during and/or analysed during the current study are available from the corresponding author on reasonable request. Requests to access these datasets should be directed to JO, jmoh@snu.ac.kr.

## Ethics statement

The studies involving human participants were reviewed and approved by Seoul National University Hospital Institutional Review Board. Written informed consent for participation was not required for this study in accordance with the national legislation and the institutional requirements.

## Author contributions

KoM: conceptualization, methodology, investigation, formal analysis, writing—original draft, writing—editing, and visualization. KimMG: conceptualization, methodology, and writing—review & editing. YoonS-S, SuhSY, and ChoY-S: resources. KimI-W: methodology and writing—review & editing. OhJM: methodology, resources, writing—review & editing, supervision, and project administration. All authors contributed to the article and approved the submitted version.

## Funding

This study was supported by the National Research Foundation of Korea grant funded by the Korea government (MSIT) (no. 2021R1A2C1006046).

## Conflict of interest

The authors declare that the research was conducted in the absence of any commercial or financial relationships that could be construed as a potential conflict of interest.

## Publisher’s note

All claims expressed in this article are solely those of the authors and do not necessarily represent those of their affiliated organizations, or those of the publisher, the editors and the reviewers. Any product that may be evaluated in this article, or claim that may be made by its manufacturer, is not guaranteed or endorsed by the publisher.

## References

[1] Paul S Kantarjian H Jabbour EJ. Adult acute lymphoblastic leukemia. Mayo Clin Proc. (2016) 91:1645–66. Epub 2016/11/07. doi: 10.1016/j.mayocp.2016.09.010 27814839

[2] Pui CH Evans WE. Treatment of acute lymphoblastic leukemia. N Engl J Med. (2006) 354:166–78. Epub 2006/01/13. doi: 10.1056/NEJMra052603 16407512

[3] Brown PA Shah B Advani A Aoun P Boyer MW Burke PW. Acute lymphoblastic leukemia, version 2.2021, Nccn clinical practice guidelines in oncology. J Natl Compr Cancer Netw. (2021) 19:1079–109. Epub 2021/09/23. doi: 10.6004/jnccn.2021.0042, PMID: 34551384

[4] Emadi A Zokaee H Sausville EA. Asparaginase in the treatment of non-all hematologic malignancies. Cancer Chemother Pharmacol. (2014) 73:875–83. Epub 2014/02/12. doi: 10.1007/s00280-014-2402-3, PMID: 24515335

[5] Boos J. Pharmacokinetics and drug monitoring of L-Asparaginase treatment. Int J Clin Pharmacol Ther. (1997) 35:96–8. Epub 1997/03/01. 9088996

[6] Ahlke E Nowak-Gottl U Schulze-Westhoff P Werber G Borste H Wurthwein G. Dose reduction of Asparaginase under pharmacokinetic and Pharmacodynamic control during induction therapy in children with acute lymphoblastic Leukaemia. Br J Haematol. (1997) 96:675–81. Epub 1997/03/01. doi: 10.1046/j.1365-2141.1997.d01-2089.x, PMID: 9074406

[7] Steiner M Hochreiter D Kasper DC Kornmuller R Pichler H Haas OA. Asparagine and aspartic acid concentrations in bone marrow versus peripheral blood during Berlin-Frankfurt-Munster-based induction therapy for childhood acute lymphoblastic leukemia. Leuk Lymphoma. (2012) 53:1682–7. Epub 2012/02/24. doi: 10.3109/10428194.2012.668681, PMID: 22356135

[8] Verma N Kumar K Kaur G Anand S. L-Asparaginase: a promising chemotherapeutic agent. Crit Rev Biotechnol. (2007) 27:45–62. Epub 2007/03/17. doi: 10.1080/07388550601173926, PMID: 17364689

[9] Jayaram HN Cooney DA Huang CY. Interaction between L-aspartic acid and L-Asparaginase from Escherichia Coli: binding and inhibition studies. J Enzym Inhib. (1986) 1:151–61. Epub 1986/01/01. doi: 10.3109/14756368609020113, PMID: 3334241

[10] Citri N Kitron N Zyk N. Stereospecific features of the Conformative response of L-Asparaginase. Biochemistry. (1972) 11:2110–6. Epub 1972/05/23. doi: 10.1021/bi00761a018, PMID: 4554898

[11] Citri N Zyk N. Stereospecificity of the catalytic reaction of L-Asparaginase. Biochemistry. (1972) 11:2103–9. Epub 1972/05/23. doi: 10.1021/bi00761a017, PMID: 4554897

[12] Zhang X Pang L Sharma SV Li R Nyitray AG Edwards BJ. Malnutrition and overall survival in older patients with cancer. Clin Nutr. (2021) 40:966–77. Epub 2020/07/16. doi: 10.1016/j.clnu.2020.06.026 32665101

[13] Bullock AF Greenley SL McKenzie GAG Paton LW Johnson MJ. Relationship between markers of malnutrition and clinical outcomes in older adults with cancer: systematic review, narrative synthesis and meta-analysis. Eur J Clin Nutr. (2020) 74:1519–35. Epub 2020/05/06. doi: 10.1038/s41430-020-0629-0, PMID: 32366995 PMC7606134

[14] Bozzetti F. Total parenteral nutrition in cancer patients. Curr Opin Support Palliat Care. (2007) 1:281–6. Epub 2008/08/08. doi: 10.1097/SPC.0b013e3282f1bf60 18685376

[15] Joque L Jatoi A. Total parenteral nutrition in cancer patients: why and when? Nutr Clin Care. (2005) 8:89–92. Epub 2005/07/15. 16013227

[16] Ravasco P. Nutrition in cancer patients. J Clin Med. (2019) 8 Epub 2019/08/17:1211. doi: 10.3390/jcm8081211 31416154 PMC6723589

[17] Oken MM Creech RH Tormey DC Horton J Davis TE McFadden ET. Toxicity and response criteria of the eastern cooperative oncology group. Am J Clin Oncol. (1982) 5:649–56. Epub 1982/12/01. doi: 10.1097/00000421-198212000-00014 7165009

[18] Raetz EA Salzer WL. Tolerability and efficacy of L-Asparaginase therapy in pediatric patients with acute lymphoblastic leukemia. J Pediatr Hematol Oncol. (2010) 32:554–63. Epub 2010/08/21. doi: 10.1097/MPH.0b013e3181e6f003, PMID: 20724951

[19] Mohamed SA Elshal MF Kumosani TA Aldahlawi AM. Purification and characterization of Asparaginase from Phaseolus Vulgaris seeds. Evid Based Complement Alternat Med. (2015) 2015:309214. Epub 2015/09/29. doi: 10.1155/2015/309214, PMID: 26413120 PMC4564614

[20] Tan IK Gajra B. Plasma and urine amino acid profiles in a healthy adult population of Singapore. Ann Acad Med Singap. (2006) 35:468–75. Epub 2006/08/12. doi: 10.47102/annals-acadmedsg.V35N7p468, PMID: 16902722

[21] Berard MP Hankard R Cynober L. Amino acid metabolism during Total parenteral nutrition in healthy volunteers: evaluation of a new amino acid solution. Clin Nutr. (2001) 20:407–14. Epub 2001/09/06. doi: 10.1054/clnu.2001.0466, PMID: 11534935

[22] Bachmann KA Lewis JD. Predicting inhibitory drug-drug interactions and evaluating drug interaction reports using inhibition constants. Ann Pharmacother. (2005) 39:1064–72. Epub 2005/05/12. doi: 10.1345/aph.1E508, PMID: 15886285

[23] Egler RA Ahuja SP Matloub Y. L-Asparaginase in the treatment of patients with acute lymphoblastic leukemia. J Pharmacol Pharmacother. (2016) 7:62–71. Epub 2016/07/22. doi: 10.4103/0976-500x.184769, PMID: 27440950 PMC4936081

[24] Vileisis RA Inwood RJ Hunt CE. Laboratory monitoring of parenteral nutrition-associated hepatic dysfunction in infants. JPEN J Parenter Enteral Nutr. (1981) 5:67–9. Epub 1981/01/01. doi: 10.1177/014860718100500167, PMID: 6785477

[25] Salvino R Ghanta R Seidner DL Mascha E Xu Y Steiger E. Liver failure is uncommon in adults receiving long-term parenteral nutrition. JPEN J Parenter Enteral Nutr. (2006) 30:202–8. Epub 2006/04/28. doi: 10.1177/0148607106030003202, PMID: 16639066

[26] Arends J Baracos V Bertz H Bozzetti F Calder PC Deutz NEP. Espen expert group recommendations for action against cancer-related malnutrition. Clin Nutr. (2017) 36:1187–96. Epub 2017/07/12. doi: 10.1016/j.clnu.2017.06.017, PMID: 28689670

